# Unraveling the mechanism of sulfur nutrition in pigeonpea inoculated with sulfur-oxidizing bacteria

**DOI:** 10.3389/fmicb.2022.927702

**Published:** 2022-09-05

**Authors:** Deepti Malviya, Ajit Varma, Udai B. Singh, Shailendra Singh, Anil K. Saxena

**Affiliations:** ^1^Amity Institute of Microbial Technology, Amity University, Noida, Uttar Pradesh, India; ^2^Plant-Microbe Interaction and Rhizosphere Biology Lab, ICAR-National Bureau of Agriculturally Important Microorganisms, Maunath Bhanjan, Uttar Pradesh, India

**Keywords:** sulfur-oxidizing bacteria, pigeon pea (*Cajanus cajan*), sulfate transporter, *PpSULTR*, root colonization, root architecture, ROS

## Abstract

An investigation was carried out to understand the mechanism(s) involved in the uptake of sulfur (S) as sulfate in pigeonpea following single inoculation of two sulfur-oxidizing bacteria (SOB), *Stenotrophomonas maltophilia* and *Stenotrophomonas pavanii* in the treatments amended with either elemental sulfur (S^0^) or sulfate (S^6^). Colonization potential and biofilm formation were analyzed through confocal laser scanning microscope (CLSM) and scanning electron microscope (SEM). Furthermore, the effect of seed inoculation on root architecture, expression of genes involved in sulfur oxidation (*sox*) in bacterial inoculants, and genes involved in sulfate transport in pigeonpea (*PpSULTR*) were analyzed to correlate with the higher uptake of S in roots and shoots of pigeonpea. Both the SOB exhibited a good colonization potential and biofilm formation on the roots of pigeonpea. Among the 11 *sox* genes targeted in rhizosphere of pigeonpea, expression was achieved for seven genes, which showed 2-fold increase in treatments inoculated with *S. maltophilia* and amended with either S^6^ or S^0^. The inoculation of *S. maltophilia* and amendment of S^0^ led to increased expression of *PpSULTR* genes by several folds in roots. The inoculation of SOB had a significant influence on non-enzymatic (osmolytes like proline) and enzymatic (PAL, peroxidase, superoxide dismutase, and catalase) levels. The results revealed a significant increase in sulfur uptake in roots and shoots in treatment inoculated with *S. maltophilia* and amended with S^6^. The investigation showed that the SOB-mediated over-expression of *PpSULTR* genes in roots of pigeonpea and *sox* genes in the rhizosphere were acting synergistically in facilitating higher uptake and translocation of S in roots and shoots of pigeonpea plants.

## Introduction

Sulfur is an important nutrient for the plant growth and development. Plants take-up sulfur (S) in the form of sulfate (S^6^), which is ^+^6 oxidation state of sulfur (Takahashi et al., 2011a,b; Giovannetti et al., 2014). In soils, sulfur is chiefly present in bound form as organic compounds (Takahashi et al., 2011a,b; Giovannetti et al., 2014). The plants utilize the oxidized form of S for the biosynthesis of S-rich amino acids such as cysteine, cystine, and methionine, glutathione, secondary metabolites, sulfoflavonoids, S-containing co-enzymes, and prosthetic groups (Giovannetti et al., 2014). In the last two decades, there are the reports of S-deficiency in different soil types across the globe. There are many factors contributing to this decline in S content in the soil. Intensive cropping patterns, low organic matter particularly in tropical soils, and extensive use of chemical fertilizers that are low in sulfur are some of the factors that influence plant growth due to poor availability of sulfur (McGrath et al., 2002; Lewandowska and Sirko, 2008). The conventional solution to this problem is the use of S-based chemical fertilizers. In general, it is recommended to apply elemental sulfur (S^0^) as compared to sulfate (S^+6^) for the proper growth and development of plants (Bouranis et al., 2018; Fuentes-Lara et al., 2019). However, there are microorganisms that have the ability to convert S^0^ to S^+6^ and are collectively termed sulfur-oxidizing bacteria (SOB) (Frigaard and Dahl, 2009; Zhi-Hui et al., 2010; Wang et al., 2019). Both autotrophic and heterotrophic sulfur-oxidizing bacteria have been isolated from different ecological niches (Rawlings, 2005; Majumder and Palit, 2017; Berben et al., 2019; Wang et al., 2019; Zhang et al., 2019). They are metabolically and nutritionally diverse, which includes autotrophs, heterotrophs and mixotrophs. The autotrophs include *Acidithiobacillus ferrooxidans, Acidithiobacillus thiooxidans, Ancylobacter aquaticus, Halothiobacillus kellyi, Mesorhizobium thiogangeticum, Methylobacterium thiocyanatum, Thiobacillus denitrificans, Thiobacillus thioparus, Thiomonas cuprina, Thiomonas intermedia, Thiomonas perometabolis*, and *Thiomonas thermosulfata* (Wood et al., 1998; Bacelar-Nicolau and Johnson, 1999; Sievert et al., 2000; Chen et al., 2004; Kumar et al., 2018, 2022). The heterotrophs include species of *Achromobacter*, *Arthrobacter*, *Brevibacterium*, *Dyella thiooxydans, Flavobacterium*, *Klebsiella*, *Micrococcus*, *Mycobacterium*, *Pandoraea thiooxydans*, *Paracoccus, Streptomyces*, *Thiosphaera*, and *Xanthobacter* (Anandham et al., 2009, 2010, 2011; Ghosh and Dam, 2009; Ryan et al., 2009; Sajjad et al., 2016; Chaudhary et al., 2017, 2021; Hou et al., 2018). However, mixotrophs include species of *Aeromonas, Alcaligenes, Bacillus*, *Bordetella, Burkholderia kururiensis subsp. Thiooxydans, Citrobacter, Diaphorobacter, Micrococcus, Pseudomonas*, *Paenibacillus, Pseudoclavibacter, Rhizobium*, and *Stenotrophomonas*, and they are the key bacterial species playing a key role in nutrient mineralization and promoting plant growth (Anandham et al., 2009; Sultan and Faisal, 2016; Malviya et al., 2022; Sanwani et al., 2022). There are many reports available on the positive influence of inoculation of these SOB on plant growth and yield (Anandham et al., 2007; Berben et al., 2019). In oil seed crops, these bacteria also help in improving the oil recovery and oil quality (Anandham et al., 2007). In legumes, the deficiency of sulfur has been reported to inhibit the process of nodulation and nitrogen fixation (Anandham et al., 2007; Cheng et al., 2017). Rhizosphere engineering of crop plants using SOB as inoculants appears to be a safe alternative to S-containing chemical fertilizers.

There are few reports available on the mechanisms by which SOB exerts its influence on uptake of S in plants. The sulfate taken-up by the plant roots is transported from roots to shoots and to seeds through various sulfate transporters. The sulfate transporters and the genes involved therein have been identified in the model plant *Arabidopsis* and a few other crop plants (Yoshimoto et al., 2007; Maruyama-Nakashita et al., 2015). In *Arabidopsis*, about 12 sulfate transporters (*SULTR*) were identified that vary in their affinity and location (Vidmar et al., 2000; Yoshimoto et al., 2002, 2003, 2007; Maruyama-Nakashita et al., 2015). A number of four groups of sulfur transporters (*SULTR1*, *SULTR2*, *SULTR3*, and *SULTR4*) have been identified that are involved in translocation of sulfate from soil to roots and in vascular translocation to other parts of the plant (Takahashi et al., 2000; Shibagaki et al., 2002; Yoshimoto et al., 2002). They are also involved in release of vacuolar sulfate to maintain sustained release and utilization of S-pools in the plant system (Kataoka et al., 2004; Maruyama-Nakashita et al., 2015).

Pigeonpea is the second most important legume grown in India and ranks sixth among the legumes globally (Varshney et al., 2012). In general, pulses are reported to have deficiency of sulfur containing amino acids (Bressani et al., 1986; Singh and Diwakar, 1993; Saxena et al., 2010) and the fulfillment of S requirement in pigeonpea is largely dependent upon the use of chemical fertilizers (Jat and Ahlawat, 2010; Kumar et al., 2012). Consequences of chemical fertilizers use include deterioration soils quality, contamination of the environment, and negative impact on human and animal health. The negative impacts of chemicals have compelled researchers and policymakers to look for alternative strategies. Among them, plant-breeding approach is one of the alternative strategies where plant breeders are targeting this issue through breeding approaches using suitable donor parents. However, the availability of the suitable donor parents and the transfer of desired traits into a suitable commercial cultivar using a backcross/marker-assisted breading program is a great challenge to the pulse breeders. Under these circumstances, the use of microbe-based strategies for S nutrition is an emerging technique/approach, which is environment-friendly and residue-free. The utilization of SOB could be an alternative approach to improve sulfur content in pigeonpea. In our earlier study, we reported strains of *Stenotrophomonas maltophilia* and *S. pavanii* isolated from different samples collected from Open Cast Projects of Jharkhand (India), to be efficient for sulfur oxidation and plant growth promotion (Malviya et al., 2022). These strains exhibited multiple plant growth-promoting traits and their inoculation enhanced the activity of reactive oxygen scavenging (ROS) enzymes and uptake of nitrogen, phosphorus, and sulfur in pigeonpea (Malviya et al., 2022). In-depth investigation is required to understand the key mechanisms playing role in the S oxidation in the rhizosphere along with S uptake and translocation in the pigeonpea. In this study, we performed a comprehensive investigation of the pigeonpea *SULTR* genes family using comparative genomics and phylogenetic analyses. Furthermore, we characterized the biofilm forming S-oxidizing microbial inoculants and attempted to explain the microbe-mediated mechanisms of S-transport in pigeonpea plants using physio-biochemical and molecular approaches. This work presents the analyses of the *SULTR* genes family, and the results will provide a basis for further investigation on the microbe-mediated modulation of *SULTR* genes for efficient uptake and translocation of sulfur in other plants.

## Materials and methods

### Bacterial strains and growth conditions

In total, two sulfur-oxidizing bacterial strains, *Stenotrophomonas maltophilia* DRC-18-7A (MZ436650) and *Stenotrophomonas pavanii* DRC-18-7B (MZ436648) previously isolated from coal mines (23°41′42.20″N 85°17′42.99″E), were obtained from Plant–Microbe Interaction and Rhizosphere Biology Lab, ICAR-National Bureau of Agriculturally Important Microorganisms, Kushmaur, Maunath Bhanjan, Uttar Pradesh, India (Malviya et al., 2022). These strains were sub-cultured and maintained on thiosulfate medium (sodium thiosulfate: 5 g, sodium carbonate: 200 mg, ammonium chloride: 100 mg, di-potassium hydrogen phosphate: 100 mg, agar: 20 g, water: 1,000 ml, bromophenol blue: 100 mg, pH 8) (Veerender et al., 2014) at 28°C for 21 days and stored at 4°C.

### In-planta assay

#### Experimental setup

Pigeonpea seeds (*cv*. Malviya 13) were obtained from Department of Genetics and Plant Breeding, Banaras Hindu University, Varanasi, India. Seeds were surface sterilized with mercuric chloride (0.1%) for 3 min followed by second sterilization with ethyl alcohol (70%) for 30 s. Thereafter, seeds were washed three times with sterile water and germinated on water agar plates. The germinated seedlings were placed in the Leonard jars filled with 500 g of sterilized river sand. The Leonard jars were inoculated with 1 ml of broth suspension (2 × 10^8^ cells ml^–1^). In total, two plants were maintained in each Leonard jar. Uninoculated jars were maintained as control. The average mean temperature and relative humidity during the experimentation were 26°C and 80%, respectively.

The Leonard jar experiment was laid out in a completely randomized block design under glasshouse conditions. The experimental set-up consisted of nine different treatments: T_1_- *Stenotrophomonas maltophilia* DRC-18-7A + sulfate compound (S^6^), T_2_- *S. maltophilia* DRC-18-7A + elemental S (S^0^), T_3_- *S. pavanii* DRC-18-7B + S^6^, T_4_- *S. pavanii* DRC-18-7B + S^0^, T_5_- *S. maltophilia* DRC-18-7A, T_6_- *S. pavanii* DRC-18-7B, T_7_- S^6^, T_8_- S^0^ and T_9_- absolute control (No inoculation, -S). Each treatment was replicated 10 times. The amount of sulfur added as S^6^ or S^0^ was 54 mg in each Leonard jar. The sulfate was added through nutrient solution, whereas elemental S was mixed with the sterile sand used to fill Leonard jars. The composition of nutrient solution with and without sulfate ions is given in Supplementary Table 1.

#### Preparation of broth and inoculation

The selected strains were inoculated in the thiosulfate broth (Veerender et al., 2014), incubated for 7 days in the incubator shaker at 150 RPM at 28°C. Broth culture of each bacterium (1 ml, 2 × 10^8^ cfu ml*^–^*^1^) was inoculated over seeds in each Leonard jar.

#### Root colonization

After 15 days of sowing, the plants from three replicates for each treatment were up-rooted gently and washed in running tap water. Confocal laser scanning microscopy was done according to the protocols described by Singh S. et al. (2020). Briefly, clean roots were treated with Syto9 and propidium iodide stains and imaging was performed using 488 and 543 nm channels under confocal scanning laser microscope (Nikon Eclipse Confocal A1, Japan). For scanning electron microscopy, root samples were washed in running tap water, fixed in mixture of formaldehyde (37%) (HiMedia, Mumbai, India) and glutaraldehyde (2.5%) (HiMedia, Mumbai, India) in 1:1 ratio for 24 h at 4°C. Thereafter, the fixed samples were kept into osmium tetroxide solution (HiMedia, Mumbai, India) for 12 h at ambient room temperature (∼27°C). The fixed root samples were dehydrated using gradient of ethyl alcohol, i.e., 30, 50, 70, 90, and 100% (30 min each) and dried under vacuum. After proper drying, the samples were coated with gold (20 nm) and visualized under scanning electron microscope (Hitachi S-3400N, United States) as described by Singh et al. (2021).

#### Effect of inoculation on plant growth attributes

After 30 days of sowing, the plants from seven replications of each treatment were uprooted. Roots were washed gently in running tap water and brought to the laboratory. The plant growth parameters such as shoot and root length and fresh and dry biomass of root and shoot were recorded.

#### Root architecture

To see the effect of seed inoculation on root architecture, roots were washed gently in running tap water and the clean roots were scanned using root scanner (Regent Instrument, Canada). The scanned images were analyzed using image analysis software “WinRhizo Pro 2017” (Client# IN1803202) and different parameters related to root architecture, secondary and tertiary rooting were recorded in the plants inoculated with selected strains and amended with S^6^ and S^0^ at 30 days of sowing.

#### Expression of genes responsible for S-oxidation in the plant rhizosphere

To evaluate the S-oxidizing activity of selected strains in the pigeonpea rhizosphere, expression analyses of genes associated with the S-oxidation were performed. For this, rhizospheric sand samples were collected from seven replicates of each treatment and brought to the laboratory in cool packs. The samples were vortexed to loosen the bacteria from sand particles. Total RNAs were isolated with the MoBio PowerSoil total RNA isolation kit (MO BIO Laboratories, Inc.) following the manufacturer’s protocols. Approximately 1 μg of RNA was used to synthesize cDNA with oligo-dT using cDNA Synthesis Kit (BioRAD, India) following the manufacturer’s instructions and quality as well as concentration of cDNA was determined using Nanodrop 2000c (Thermo Fisher Scientific, United States). For gene expression analysis, a semi-quantitative PCR method was used. The expression of genes related to S-oxidation, that is, *soxB, tetH, sdoA, sdoB, tsdA, TQO*, and *sorAB* was analyzed using gene-specific primers (Supplementary Table 2). Gene *rpoD* was taken as internal control. The final gene product obtained with RT-PCR was separated by electrophoresis in 1.5% agarose gel in TAE buffer (Mini gel electrophoresis unit, Bangalore GeNei, India), and visualization was done with the help of gel documentation system (Bio-Rad, India).

### Microbe-mediated mechanisms of sulfate uptake and translocation

#### Identification and phylogenetic analyses of sulfate transporters (*SULTRs*) in pigeonpea

Nucleotide and protein sequences of sulfate transporters (*SULTRs*) of Arabidopsis (*Arabidopsis thaliana*), soybean (*Glycin max*), field pea (*Pisum sativum*), rice (*Oryza sativa*), and wheat (*Triticum durum*) were retrieved from National Center for Biotechnology Information (NCBI) database (Supplementary Table 3). These sequences were used to search the homologous sequences in pigeonpea genome using nucleotide BLAST (Basic Local Alignment Search Tool), BLASTx (translated nucleotide → protein), tBLASTn (protein → translated nucleotide) program of NCBI. These sequences were analyzed to confirm the presence of the SULTR domain in retrieved pigeonpea homologs SULTRs gene sequences using the SMART program. Furthermore, ExPasy website^1^ was used to analyze and confirm the primary structure of SULTR proteins and several other parameters such as molecular weight, length, total number of atoms extinction coefficients, isoelectric point, aliphatic index, instability index, grand average of hydropathicity, etc. The phylogenetic tree was constructed based on the alignment of SULTR domains of pigeonpea, Arabidopsis, soybean, field pea, rice, and wheat to elucidate the phylogenetic relationships and classified them into different groups. For this, MEGA X version was used to prepare the phylogenetic tree, and neighbor-joining method was adopted with 1,000 bootstrap replications. Furthermore, primers were designed for qPCR analyses using Primer3 (v. 0.4.0) online software^2^ (Supplementary Table 4) and validated *in silico* using primer-BLAST online tools of NCBI^3^ against pigeonpea transcript sequences (*Cajanus cajan* taxid:3821).

#### Expression analysis of *PpSULTR* genes

The quantitative RT-PCR analysis was performed to investigate the expression of genes involved in sulfur uptake and transport in pigeonpea plant under different treatments. After 30 days of sowing, plants from four replications were harvested and divided into roots and shoots. The root and shoot samples were quick-frozen in liquid nitrogen, ground and total RNAs was extracted using RNA isolation kit (Agilent, India) using the manufacturer’s protocols. The cDNA was made as discussed in the previous sections “Expression of genes responsible for S-oxidation in the plant rhizosphere.” The quality and quantification of cDNA was carried out using nanodrop. The housekeeping gene actin was used as an endogenous standard to normalize the quantitative expression data. The expression of *PpSULTR* genes was analyzed using gene-specific primers designed for the present investigation (Supplementary Table 4). The qRT-PCR was performed using the SYBR Green Master Mix (Thermo Fisher Scientific) on the BioRAD Real Time PCR System (MJ MiniOpticon, BioRAD). The specificity of the amplification was verified by melting-curve analysis. The relative transcript levels were calculated using the 2^–ΔΔCT^ method (Livak and Schmittgen, 2001).

#### Effect of inoculation on physio-biochemical parameters and antioxidant enzymes

A quantitative estimation was done to evaluate the impact of inoculation of SOB on physio-biochemical properties and antioxidant enzymes in the pigeonpea leaves at 30 days of sowing. The total chlorophyll, carotenoids, total soluble sugar, and total protein in the plant leaves were measured (Sadasivam and Manickam, 1996). The accumulation of proline, phenolics, flavonoids, and superoxide dismutase (SOD) in the plant leaves was analyzed according to the procedure described by Thimmaiah (2012). The activities of PAL, peroxidase and catalase were estimated in the plant leaves according to Sadasivam and Manickam (1996).

Histological studies were also carried out to visualize the deposition of superoxide radicals (O_2_^–^) in the leaves and program cell death. Plant leaves were sampled randomly from each treatment and used for microscopic localization of superoxide radicals (O_2_^–^) using nitroblue tetrazolium (NBT; HiMedia, India) as per the methods described by Rao and Davis (1999), and it was visualized as blue color spots on the leaves. Program cell death (PCD) was examined using Evans Blue staining as described by Baker and Mock (1994).

#### Effects of bacterial inoculation on phenylpropanoid pathway

Sequences of key genes regulating the phenylpropanoid cascade in pigeonpea were retrieved from NCBI. Primers were designed for qPCR analyses and validated *in silico* (Supplementary Table 5). The nine key genes analyzed were as follows: phenylalanine ammonia-lyase [EC:4.3.1.24], phenylalanine/tyrosine ammonia-lyase [EC:4.3.1.25], 4-coumarate-CoA ligase [EC:6.2.1.12], cinnamoyl-CoA reductase [EC:1.2.1.44], cinnamyl-alcohol dehydrogenase [EC:1.1.1.195], peroxiredoxin 6 [EC:1.11.1.7], Ferulate-5-hydroxylase [EC:1.14.-.-], caffeoyl-CoA O-methyltransferase [EC:2.1.1.104], and coniferyl-aldehyde dehydrogenase [EC:1.2.1.68]. qRT-PCR analyses were performed to estimate the transcript and expression analyses (as mentioned in the previous section: Expression analysis of *PpSULTR* genes). Actin was taken as internal control.

#### Effects of inoculation on individual phenolics and flavonoids

Phenolics and flavonoids such as gallic acid, ferulic acid, sinapic acid, syringic acid, rutin, and quercetin in the plant leaves were analyzed through HPLC (binary pump model 515, 2414 refractive index (RI), and 2998 photodiode array (PDA) detector; Supelco C-18 column; Waters Pvt. Ltd.). Leaf samples (1 g) were collected from each treatment and cleaned before processing using running tap water. Active principles were extracted using methanol and acetonitrile and individual phenolics and flavonoids were measured as per the methods described by Tiwari et al. (2011).

#### Estimation of sulfate uptake

The total sulfur in plant samples was estimated using barium sulfate turbidimetry method (Garrido, 1964). In principle, during wet digestion of plant samples, sulfur present in the plants tissue is converted into sulfate ions and precipitated as barium sulfate after treatment with barium chloride. Briefly, 1 g of plant sample was taken in a 100-ml Erlenmeyer flask and pre-digested for 8 h using 10 ml of concentrated HNO_3_. The samples were further digested by addition of 10 and 3 ml of HCIO_4_ (3 ml) in flasks. The flasks were placed on a hot plate, heated at 100°C for 1 h, and subsequently, the temperature was raised to 200°C. The heating was continued until the contents became colorless and reduced to 3 ml. The flasks were cooled at room temperature. Approximately 1 ml HCl (6*N*) and 1 ml Gum acacia (0.5%) were added and mixed properly by swirling, and finally, 0.5 g BaCl_2_.2H_2_O crystals were added to the flasks. The samples were mixed until BaCl_2_.2H_2_O crystals were dissolved completely. The reading was taken at 420 nm using UV-Vis spectrophotometer. The S-content in the plant samples was calculated using the reading of standards.

### Statistical analysis

The data were subjected to the analysis of variance and least significant difference (LSD) at *p* ≤ 0.05 using SPSS 16.0. Data were compared with Duncan’s multiple range test at *p* ≤ 0.05. Graphs were prepared using statistical software Origin (Version 9) and Microsoft Office Excel (2010).

## Results

In this study, the microbe-mediated mechanisms of S-oxidation and enhanced uptake and translocation of sulfate ions in the pigeonpea at the early stage of crop growth were elucidated.

### Root colonization

**FIGURE 1:**
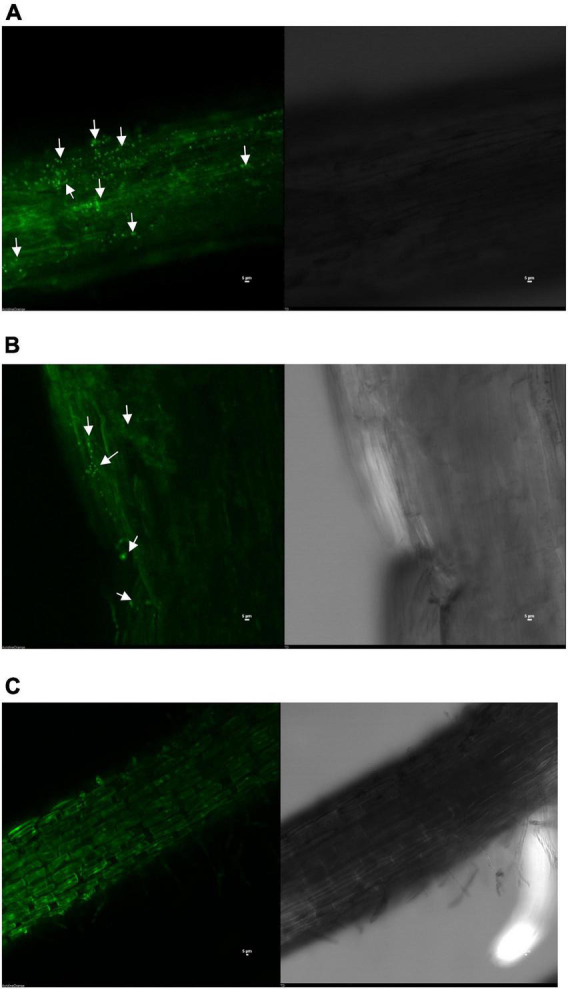
Confocal microphotograph showing root colonization by *S. maltophilia* DRC-18-7A **(A)**, *S. pavanii* DRC-18-7B **(B)** and uninoculated control **(C)**.

Confocal laser scanning microscopic and scanning electron microscopic photographs clearly showed that both the strains have the potential to colonize and develop biofilm on pigeonpea roots even under limited S-availability at 15 days of sowing. The colonization pattern/efficiency was different for the two strains on root surface. Confocal microphotograph clearly indicated that strain *S. maltophilia* DRC-18-7A produced primarily micro-aggregates and later on converted into macro-aggregates on the root surface after 15 days of inoculation (Figure 1A). Microphotograph of *S. pavanii* DRC-18-7B-treated roots revealed primarily single cells embedded in the root epidermis and rarely formed micro-aggregates (Figure 1B). However, no such evidence of bacterial colonization was observed in untreated control plants (Figure 1C).

**FIGURE 2:**
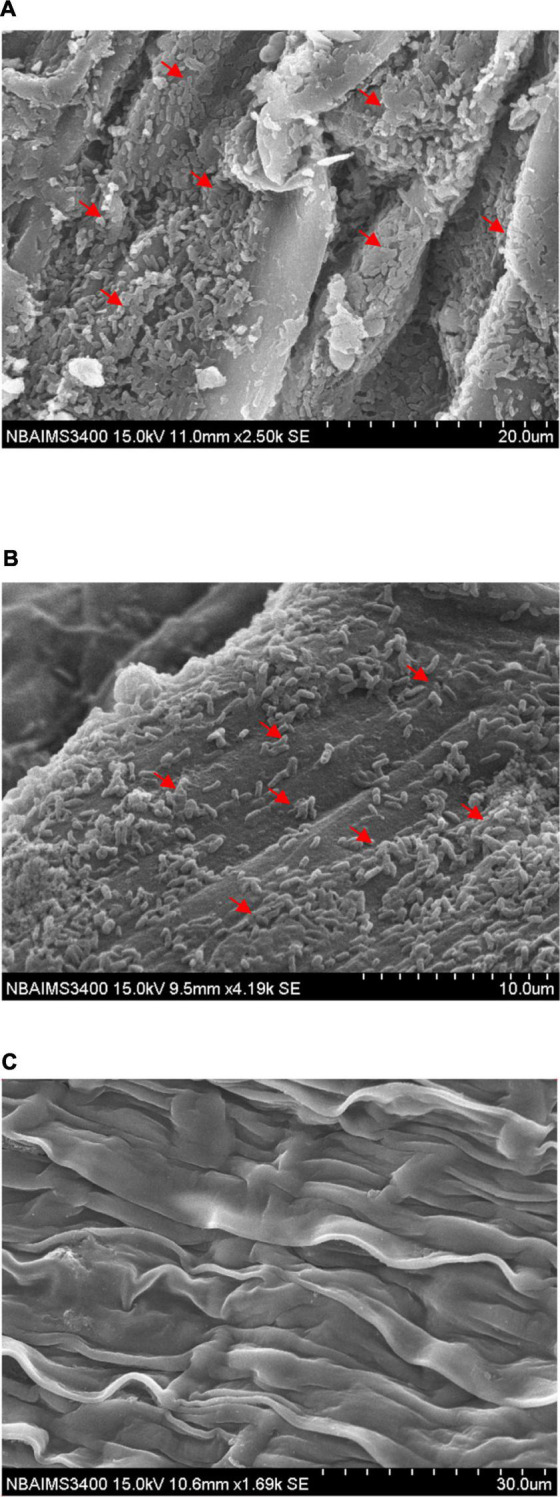
Scanning electron microphotographs showing root colonization by *S. maltophilia* DRC-18-7A **(A)**, *S. pavanii* DRC-18-7B **(B)**, and uninoculated control **(C)**.

*Stenotrophomonas maltophilia* DRC-18-7A colonized pigeonpea roots at a very high population density which is clearly visible in scanning electron microphotographs where cells were anchored to the root surfaces and to themselves by a network of fibrillar material, exo-polysaccharide produced by them on the root surface (Figure 2A). It is clearly visible in the scanning electron microphotograph that strain *S. maltophilia* DRC-18-7A produced ample amount of exo-polysaccharide and formed microbiotic crust on the root surface and bacterial cells were embedded/trampled in the crust on the root surface. In general, *S. maltophilia* DRC-18-7A cover entire root and produced thick biofilm by forming micro-aggregates and macro-aggregates (Figure 2A). From scanning electron microphotograph of *S. pavanii* DRC-18-7B, it is clear that strain *S. pavanii* DRC-18-7B is a better root colonizer (Figure 2B). *S. pavanii* DRC-18-7B population was spread on the entire root by forming micro-aggregates, and sometime, single-single cells are visible. In contrast, it is not producing too much of exo-polysaccharides as compared to *S. maltophilia* DRC-18-7A (Figure 2B). However, no such evidence of bacterial colonization was observed in untreated control plants (Figure 2C).

### Effects of inoculation on plant growth attributes at early stage

Inoculation with *S. maltophilia* DRC-18-7A and *S. pavanii* DRC-18-7B significantly enhanced the plant growth attributes in pigeonpea both in the presence of S^6^ and S^0^. In general, all the growth parameters recorded (root and shoot length, root and shoot fresh weight, root and shoot dry weight) were significantly higher in treatment inoculated with *S. maltophilia* and amended with SO_4_^2–^ compound (Table 1).

**TABLE 1:** Effects of inoculation on plant growth attributes in pigeonpea at 30 days of sowing under glasshouse conditions.

Treatments	Shoot length (cm)	Root length (cm)	Shoot fresh wt. (g)	Root fresh wt. (g)	Shoot dry wt. (g)	Root dry wt. (g)
T_1_- *S. maltophilia* DRC-18-7A + Sulfate compound	24.95	19.66	5.76	1.84	1.50	0.66
T_2_- *S. maltophilia* DRC-18-7A + Elemental S	21.37	16.50	3.95	1.33	1.25	0.50
T_3_- *S. pavanii* DRC-18-7B + Sulfate compound	21.46	17.35	5.50	1.66	1.35	0.50
T_4_- *S. pavanii* DRC-18-7B + Elemental S	19.33	14.20	3.25	1.26	1.20	0.40
T_5_- *S. maltophilia* DRC-18-7A	16.20	10.50	3.50	1.10	1.25	0.26
T_6_- *S. pavanii* DRC-18-7B	15.45	9.66	3.25	1.05	1.20	0.28
T_7_- Sulfate compound	18.75	14.26	3.96	0.98	1.10	0.36
T_8_- Elemental S	15.30	10.25	2.25	0.86	0.75	0.25
T_9_- untreated control (-S)	14.05	8.10	1.95	0.76	0.66	0.20
**CD at 5%**	**1.50**	**1.05**	**0.25**	**0.10**	**0.12**	**0.08**

### Root architecture

The inoculation of SOB along with sulfate compound significantly enhanced the root parameters as analyzed through root scanner as compared to all other treatments (Table 2). Among the two strains, inoculation of *S. maltophilia* DRC-18-7A significantly influenced root architecture in the presence of both sulfate and elemental sulfur. Treatments with only inoculation of SOB or only amendment of SO_4_^2–^ or S^0^ could not significantly influence the root parameters compared to absolute control (no inoculation and no S amendment) (Table 2).

**TABLE 2:** Effects of inoculation on root architect and root development in pigeonpea leaves at 30 days of sowing under glasshouse conditions.

Treatments	Surface area (cm^2^)	Projected area (cm^2^)	Root length (cm)	Length per volume (cm/m^3^)	Average diameter (mm)	Lateral total length (cm)	Tertiary N axis	Tertiary total length (cm)	Number of tips	Number of forks	Number of crossings	Number of links
T_1_- *S. maltophilia* DRC-18-7A + Sulfate compound	9.12	2.90	45.39	57.34	0.70	29.25	76.29	29.12	78.29	139.50	18.96	256.10
T_2_- *S. maltophilia* DRC-18-7A + Elemental S	6.97	2.33	34.29	40.19	0.60	21.50	42.50	21.47	59.25	115.29	11.75	186.10
T_3_- *S. pavanii* DRC-18-7B + Sulfate compound	8.05	2.56	40.10	57.34	0.61	24.10	66.05	24.25	74.50	120.10	14.35	220.50
T_4_- *S. pavanii* DRC-18-7B + Elemental S	6.86	2.30	30.10	36.33	0.56	18.75	42.33	16.25	48.10	104.10	10.25	166.25
T_5_- *S. maltophilia* DRC-18-7A	5.25	1.95	26.75	39.26	0.50	18.25	40.35	20.50	55.25	110.23	8.35	190.33
T_6_- *S. pavanii* DRC-18-7B	5.10	1.75	25.66	38.10	0.49	17.33	38.10	20.66	52.33	100.35	9.03	180.34
T_7_- Sulfate compound	5.74	2.10	29.52	40.50	0.52	20.33	58.10	18.05	58.29	116.05	10.50	192.33
T_8_- Elemental S	4.25	1.65	25.10	27.25	0.49	14.25	30.50	10.50	35.78	76.67	6.67	140.25
T_9_- untreated control (-S)	3.84	1.33	24.66	20.10	0.28	10.10	21.15	8.05	29.15	60.50	4.26	121.05
**CD at 05%**	**0.62**	**0.20**	**1.75**	**2.03**	**0.02**	**1.05**	**1.84**	**1.50**	**3.67**	**2.97**	**1.74**	**4.65**

### Expression of genes responsible for S-oxidation in the plant rhizosphere

**FIGURE 3:**
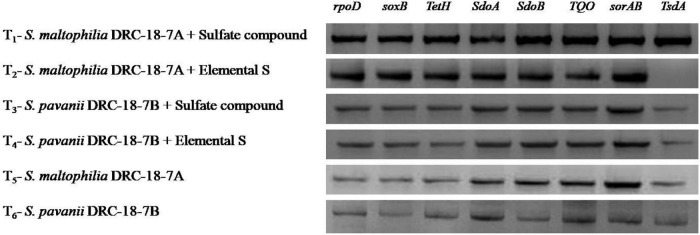
Effects of different treatments on expression of genes responsible for S-oxidation in the plant rhizosphere, treatments were as follows: T_1_-*Stenotrophomonas maltophilia* DRC-18-7A + Sulfate compound, T_2_-*S. maltophilia* DRC-18-7A + Elemental S, T_3_-*S. pavanii* DRC-18-7B + Sulfate compound, T_4_-*S. pavanii* DRC-18-7B + Elemental S, T_5_-*S. maltophilia* DRC-18-7A, T_6_-*S. pavanii* DRC-18-7B.

Among the 11 sox genes targeted in rhizosphere of pigeonpea, expression was achieved for 7 genes (*soxB, tetH, sdoA, sdoB, TQO, sorAB*, and *tsdA*), which showed 2-fold increase in treatments inoculated with *S. maltophilia* and amended with either S^6^ or S^0^. Similar tends were not observed in respective treatments inoculated with *S. pavanii* (Figure 3). The results revealed significantly higher transcript accumulation for genes *sdoB, TQO, sorAB*, and *tsdA* in the rhizosphere of plants inoculated with *S. maltophilia* (T-1). In general, expression and transcript accumulation of genes *tsdA, soxB*, and *tetH* were significantly lower across the treatments as compared to other genes.

### Identification of *SULTRs* gene in the pigeonpea and *in silico* validation

**FIGURE 4:**
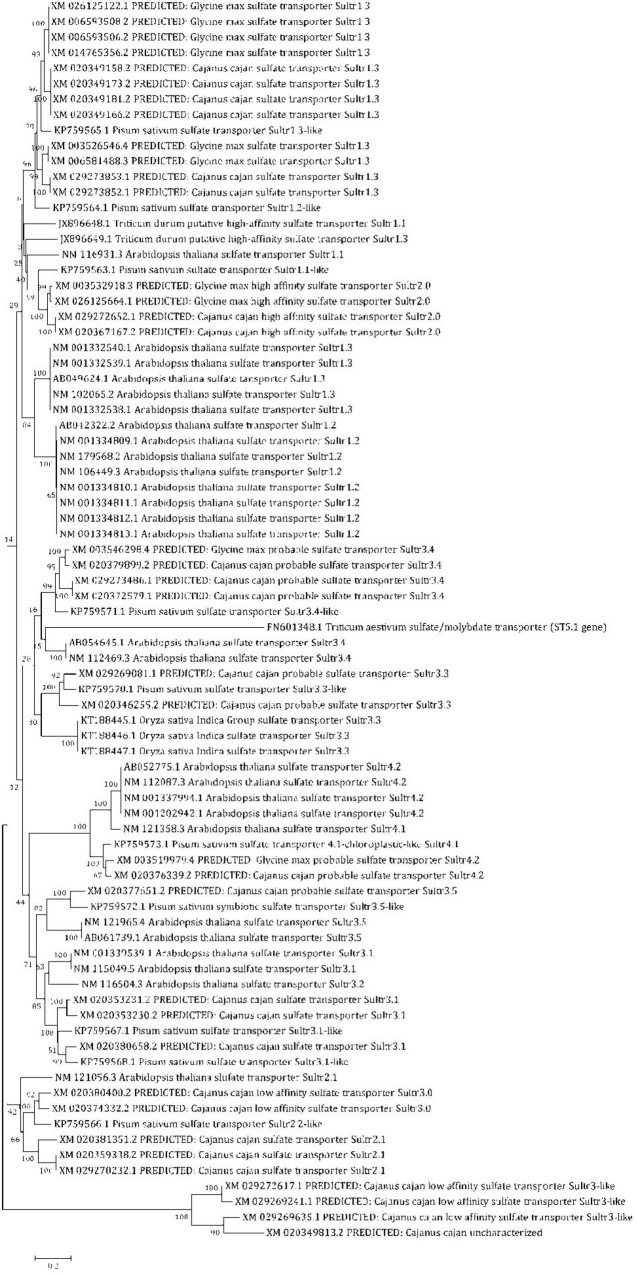
Phylogenetic tree showing the relationships of SULTR domains of pigeonpea, with other crop plants, Arabidopsis, soybean, field pea, rice, and wheat and classified into different groups.

For identification of *SULTR* genes in pigeonpea, 10 *AtSULTRs*, 4 *GmSULTRs*, 9 *PsSULTRs*, 1 *OsSULTRs*, and 3 *TdSULTRs* were used as query sequences for BLASTn searches of the pigeonpea database (*Cajanus cajan*, taxid:3821) in NCBI with default parameters and redundant sequences were discarded manually. As a result, 11 SULTR genes, i.e., *PpSULTR1.1, PpSULTR1.2, PpSULTR1.3, PpSULTR2.1, PpSULTR2.2, PpSULTR3.1, PpSULTR3.3, PpSULTR3.3-like, PpSULTR3.4, PpSULTR3.5, PpSULTR4.1*, and *PpSULTR4.2* were identified in the pigeonpea genome. These putative SULTR genes are located on different chromosomes. Their proteins contain STAS domain and the C-terminal region, which are critical for sulfate transporter activity and stability. To gain insights into the biological function of these genes and close relatives, a phylogenetic tree was constructed based on the full-length amino acid sequence alignment of SULTRs including 26 putative pigeonpea *SULTR* sequences, 10 *AtSULTRs*, 4 *GmSULTRs*, 9 *PsSULTRs*, 1 *OsSULTRs*, and 3 *TdSULTRs* (Figure 4). Based on phylogeny, the *PpSULTRs* are closely related to soybean *SULTRs* (*GmSULTRs*) and classified into four groups based on phylogenetic analyses. These pigeonpea *SULTR* genes were named corresponding to the homologous genes from other species.

To validate the reliability of the expression profile, *in silico* PCR amplification as well as validation was done using genomic sequences of pigeonpea as query sequence. Based on the *in silico* amplification, a set of primers were selected for real-time gene expression analysis in the pigeonpea grown under different treatments.

### Effects of inoculation on expression of *SULTR* genes in pigeonpea

**FIGURE 5:**
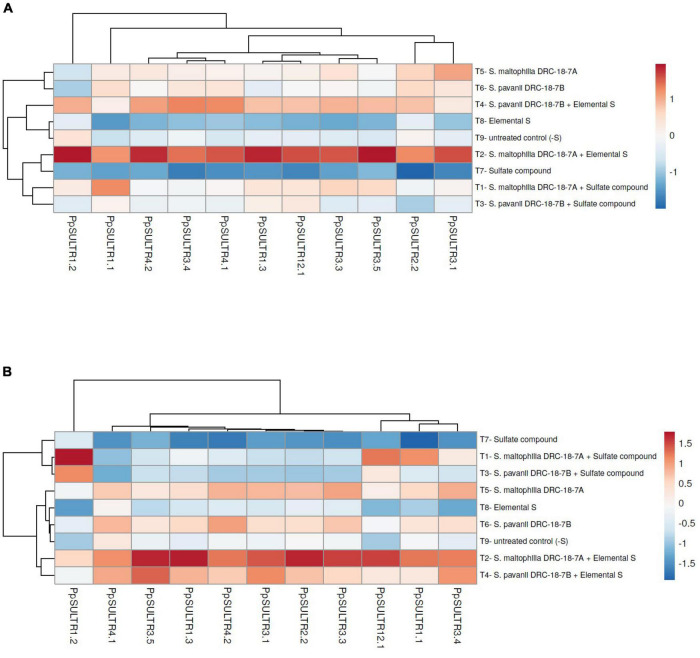
Heatmap showing the effects of microbial inoculation on expression of *SULTR* genes in pigeonpea **(A)** root and **(B)** shoot at 30 days of sowing T_1_-*Stenotrophomonas maltophilia* DRC-18-7A + Sulfate compound, T_2_-*S. maltophilia* DRC-18-7A + Elemental S, T_3_-*S. pavanii* DRC-18-7B + Sulfate compound, T_4_-*S. pavanii* DRC-18-7B + Elemental S, T_5_-*S. maltophilia* DRC-18-7A, T_6_-*S. pavanii* DRC-18-7B, T_7_-Sulfate compound, T_8_-Elemental S, and T_9_-untreated control (-S).

Transcript profiling of the *PpSULTR* genes was done in the pigeonpea plants inoculated with *S. maltophilia* DRC-18-7A and *S. pavanii* DRC-18-7B. It was found that sulfur sources and microbial inoculation significantly influenced the expression profile of *PpSULTR* genes in pigeonpea which was also evident from sulfur content in pigeonpea root and shoot. Furthermore, the expression profiles of *PpSULTRs* also varied in root and shoot of the same plant. Significantly higher expression (upregulation) of all the 11 *PpSULTR* genes was recorded in the roots and shoots of pigeonpea inoculated with *S. maltophilia* DRC-18-7A and amended with elemental sulfur (Figure 5A). Likewise, the expression of these genes in the roots and shoots of plants from treatment inoculated with *S. pavanii* and amended with S^0^ was higher and the fold increase closely followed treatment with *S. maltophilia* + S^0^. In general, the expression levels of *PpSULTR* genes in the roots were significantly higher (3–5-folds) as compared to the shoots. Interestingly, it was found that the expression level (fold change) of *PpSULTRs* was slightly higher in the negative control (-S) as compared to positive control (+S) (Figure 5B).

### Effects of inoculation on physio-biochemical property and antioxidant enzymes

**FIGURE 6:**
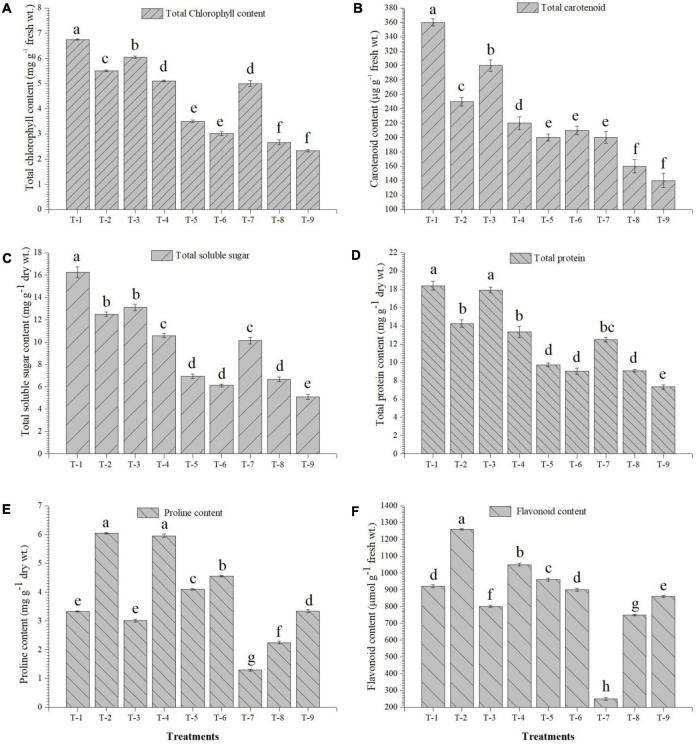
Effects of seed treatment on accumulation of **(A)** total chlorophyll, **(B)** total carotenoids, **(C)** total soluble sugar, **(D)** total protein **(E)** proline, and **(F)** flavonoids in the pigeonpea leaves at 30 days of sowing Treatments were as follows: T_1_-*Stenotrophomonas maltophilia* DRC-18-7A + Sulfate compound, T_2_-*S. maltophilia* DRC-18-7A + Elemental S, T_3_-*S. pavanii* DRC-18-7B + Sulfate compound, T_4_-*S. pavanii* DRC-18-7B + Elemental S, T_5_-*S. maltophilia* DRC-18-7A, T_6_-*S. pavanii* DRC-18-7B, T_7_-Sulfate compound, T_8_-Elemental S and T_9_-untreated control (-S). Data are mean (*n* = 10) and vertical bar represents standard deviation. Data with different letters show significant difference in column data in randomized block design test at *p* < 0.05 under Duncan’s multiple range test.

**FIGURE 7:**
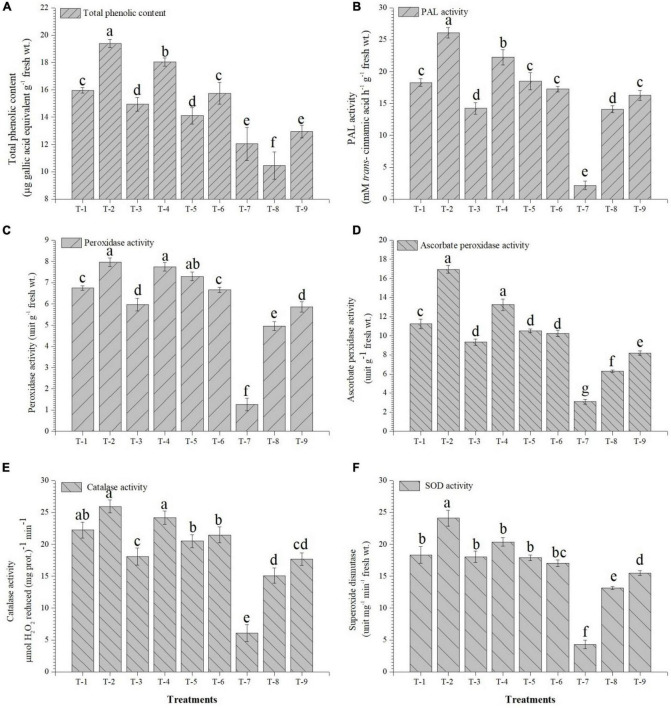
Effects of seed treatment on activities and accumulation of antioxidant biomolecules and enzymes **(A)** total phenolic, **(B)** PAL, **(C)** peroxidase, **(D)** ascorbate peroxidase **(E)** catalase, and **(F)** superoxide dismutase in the pigeonpea leaves at 30 days of sowing treatments were: T_1_-*Stenotrophomonas maltophilia* DRC-18-7A + Sulfate compound, T_2_-*S. maltophilia* DRC-18-7A + Elemental S, T_3_-*S. pavanii* DRC-18-7B + Sulfate compound, T_4_-*S. pavanii* DRC-18-7B + Elemental S, T_5_-*S. maltophilia* DRC-18-7A, T_6_-*S. pavanii* DRC-18-7B, T_7_-Sulfate compound, T_8_-Elemental S and T_9_-untreated control (-S). Data are mean (*n* = 10) and vertical bar represents standard deviation. Data with different letters show significant difference in column data in randomized block design test at *p* < 0.05 under Duncan’s multiple range test.

The inoculation of the selected strains, *S. maltophilia* DRC-18-7A and *S. pavanii* DRC-18-7B, modulated the physio-biochemical pathways and accumulation of antioxidants in the pigeonpea plants. The quantitative analysis revealed that the accumulation of total chlorophyll, carotenoids, soluble sugars, and protein content was significantly enhanced in the treatment inoculated with *S. maltophilia* and supplemented with sulfate compound (Figure 6). Inoculation of SOB alone could not influence the accumulation and was significantly lower than treatment amended with S^6^ compound. In contrast, the accumulation of proline, flavanoids, total phenolics, and activities of antioxidant enzymes (PAL, POx, APx, catalase, and SOD) were significantly enhanced in the treatment inoculated with either of the SOB and amended with elemental sulfur. The presence of S^6^ in the treatments with or without inoculation led to significantly lower accumulation of proline and flavonoids (Figure 7).

**FIGURE 8:**
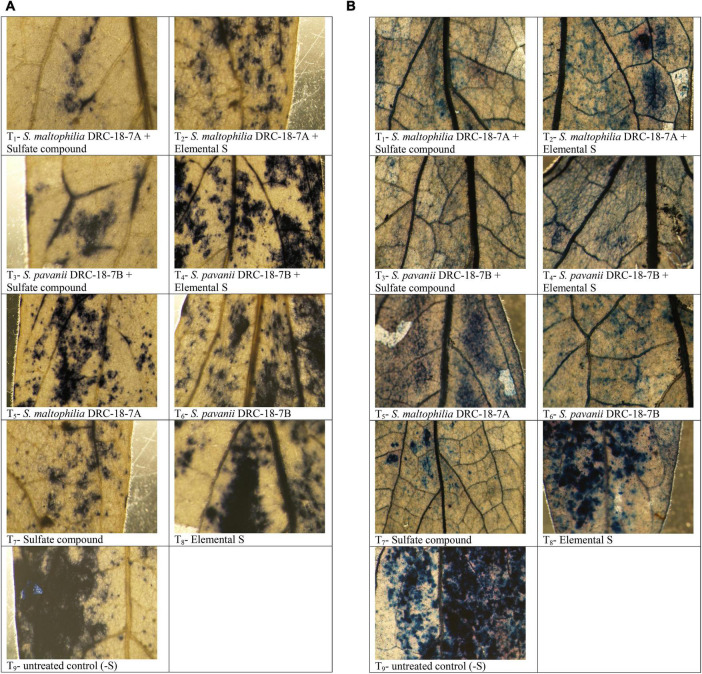
Microscopic detection of superoxide radical by NBT staining **(A)** and program cell death **(B)** in leaves of pigeonpea after treatment with T_1_-*Stenotrophomonas maltophilia* DRC-18-7A + Sulfate compound, T_2_-*S maltophilia* DRC-18-7A + Elemental S, T_3_-*S. pavanii* DRC-18-7B + Sulfate compound, T_4_-*S. pavanii* DRC-18-7B + Elemental S, T_5_-*S. maltophilia* DRC-18-7A, T_6_-*S. pavanii* DRC-18-7B, T_7_-Sulfate compound, T_8_-Elemental S and T_9_-untreated control (-S) at 30 days of sowing.

Attenuation of superoxide levels was observed as a blue formazan, which is the outcome of NBT dye and superoxide interactions (Figure 8A). Stereoscopic visualization clearly showed dense localization of superoxide radicals in the petioles near veins and midrib of leaves of the untreated control plant (negative control) followed by plants grown with elemental S. Least accumulation of superoxide radical was observed in the plants inoculated with either of strains and supplemented with sulfate compounds compared to all other treatments (Figure 8A). Similarly, program cell death was observed as greenish polymerization product of Evans Blue stain. The bacterial inoculation and supplementation of sulfate compound in the nutrient solution significantly reduced greenish discoloration compared to other treatments. Similar to superoxide radicals, maximum program cell death was observed in the untreated control plants (Figure 8B).

### Inoculation modulate expression profile of key genes of phenylpropanoid pathways

**FIGURE 9:**
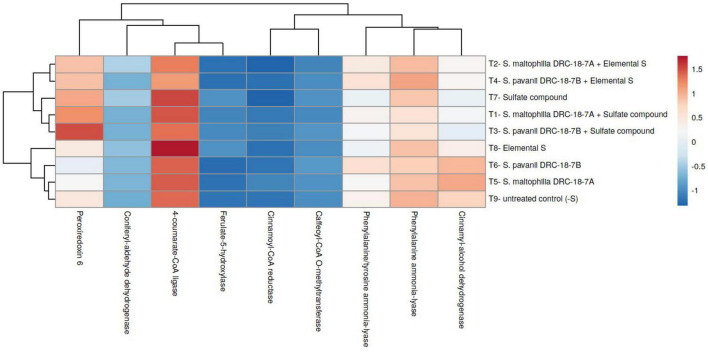
Heatmap showing the effects of microbial inoculation on expression profile of key genes of phenylpropanoid pathways in leaves of pigeonpea at 30 days of sowing T_1_- *Stenotrophomonas maltophilia* DRC-18-7A + Sulfate compound, T_2_- *S. maltophilia* DRC-18-7A + Elemental S, T_3_- *S. pavanii* DRC-18-7B + Sulfate compound, T_4_- *S. pavanii* DRC-18-7B + Elemental S, T_5_- *S. maltophilia* DRC-18-7A, T_6_- *S. pavanii* DRC-18-7B, T_7_- Sulfate compound, T_8_- Elemental S and T_9_- untreated control (-S).

The up-/downregulation of nine key genes (phenylalanine ammonia-lyase [EC:4.3.1.24], phenylalanine/tyrosine ammonia-lyase [EC:4.3.1.25], 4-coumarate-CoA ligase [EC:6.2.1.12], cinnamoyl-CoA reductase [EC:1.2.1.44], cinnamyl-alcohol dehydrogenase [EC:1.1.1.195], peroxiredoxin 6 [EC:1.11.1.7], ferulate-5-hydroxylase [EC:1.14.-.-], caffeoyl-CoA O-methyltransferase [EC:2.1.1.104], and coniferyl-aldehyde dehydrogenase [EC:1.2.1.68]) involved in phenylpropanoid pathway was investigated. The results revealed that these genes were upregulated in treatment inoculated with *S. maltophilia* DRC-18-7A and supplemented with elemental S in the leaves of pigeonpea. The highest expression of 4-coumarate-CoA ligase [EC: 6.2.1.12] was recorded in the leaves of pigeonpea plants across the treatments taken into consideration followed by phenylalanine ammonia-lyase [EC:4.3.1.24] and phenylalanine/tyrosine ammonia-lyase [EC:4.3.1.25]. However, in the inoculated plants, expression level was significantly higher in comparison with untreated positive and negative control plants (Figure 8). In contrast, comparatively less expression was recorded in the plants harvested from treatments amended with S^6^ compounds (Figure 9) as compared to plants grown with elemental S. It revealed that plants grown in the presence of S^0^ experienced stress and tend to over-express antioxidant genes.

### Effects of inoculation on individual phenolics and flavonoids

The accumulation of phenolics (gallic, ferrulic, sinapic, and syringic acids) and flavonoids (rutin and quercetin) was differentially influenced by inoculation of SOB and supplementation of two different sources of sulfur (S^6–^ or S^0^). In treatments inoculated with either *S. maltophilia* or *S. pavanii* and amended with S^0^, the levels of all analyzed phenolics and flavonoids were significantly higher than all other treatments. Addition of S^6^ compound to SOB inoculated treatments did not significantly influence the synthesis of phenolics acids and flavonoids (Table 3).

**TABLE 3:** Effects of inoculation on individual phenolic and flavonoids content in pigeonpea leaves at 30 days of sowing under glasshouse conditions.

Treatments	Gallic acid (μg g^–^^1^ fresh wt.)	Ferulic acid (μg g^–^^1^ fresh wt.)	Sinapic acid (μg g^–^^1^ fresh wt.)	Syringic acid (μg g^–^^1^ fresh wt.)	Rutin (μg g^–^^1^ fresh wt.)	Quercetin (μg g^–^^1^ fresh wt.)
T_1_- *S. maltophilia* DRC-18-7A + Sulfate compound	119.25	12.39	8.92	39.29	36.25	25.90
T_2_- *S. maltophilia* DRC-18-7A + Elemental S	145.29	17.39	13.96	62.96	56.97	22.96
T_3_- *S. pavanii* DRC-18-7B + Sulfate compound	110.33	10.03	8.05	34.10	33.33	20.75
T_4_- *S. pavanii* DRC-18-7B + Elemental S	136.10	16.05	13.05	60.50	50.10	22.50
T_5_- *S. maltophilia* DRC-18-7A	126.50	10.60	6.66	32.50	30.50	13.25
T_6_- *S. pavanii* DRC-18-7B	122.35	11.05	6.50	31.96	32.50	13.05
T_7_- Sulfate compound	69.10	4.26	3.50	10.25	6.29	4.26
T_8_- Elemental S	110.39	8.50	5.10	16.55	17.50	7.10
T_9_- untreated control (-S)	121.25	10.26	6.25	29.50	28.10	10.25
**CD at 05%**	**2.75**	**1.67**	**1.12**	**2.50**	**3.45**	**1.05**

### Sulfur uptake

The uptake of sulfur in the roots and shoots was significantly influenced by inoculation of SOB and supplementation of sulfur in different forms (Table 4). In general, for all the treatments, the S content was higher in roots as compared to shoots. Among the treatments, maximum S content was recorded due to the inoculation of *S. maltophilia* and supplementation of S^6^ compound. The S-content was 48 and 42% higher in roots and shoots, respectively, as compared to treatment where only S^6^ compound was supplemented. Similar trend was observed in treatment inoculated with *S. pavanii*. The S-content in roots and shoots of plants from treatments inoculated with SOB and supplemented with elemental S was significantly lower as compared to other treatments (Table 4).

**TABLE 4:** Effects of inoculation on sulfur content in pigeonpea at 30 days of sowing under glasshouse conditions.

Treatments	Sulfur content in shoot (g kg^–^^1^ dry wt.)	Sulfur content in root (g kg^–^^1^ dry wt.)
T_1_- *S. maltophilia* DRC-18-7A + Sulfate compound	5.05	6.25
T_2_- *S. maltophilia* DRC-18-7A + Elemental S	3.15	3.94
T_3_- *S. pavanii* DRC-18-7B + Sulfate compound	4.50	5.86
T_4_- *S. pavanii* DRC-18-7B + Elemental S	2.95	3.05
T_5_- *S. maltophilia* DRC-18-7A	0.11	0.12
T_6_- *S. pavanii* DRC-18-7B	0.10	0.10
T_7_- Sulfate compound	3.55	4.20
T_8_- Elemental S	1.19	1.50
T_9_- untreated control (-S)	0.10	0.15
**CD at 05%**	**0.45**	**0.59**

## Discussion

Besides the importance of three major nutrients, i.e., nitrogen, phosphorus, and potassium, the focus of research work has been shifted to investigate the key role of other macro- and micro-nutrients in major crop plants including pulses. The intensive agriculture has led to the deficiency of these nutrients such as S, Bo, Zn, and Fe. In the last two decades, the losses in crop yield due to the deficiency of these nutrients are now reported often from different parts of the world. Sulfur nutrition is important as it influences different metabolic pathways as a structural component of many secondary metabolites, vitamins, amino acids, and enzymes. Among the crop plants, legumes are strikingly affected by deficiency of S in soil (Chandler et al., 1984; Scherer, 2001). Besides influencing the plant growth, the process of nitrogen fixation and nodulation is hampered due to sulfur deficiency in soil (Watkinson, 1989; Scherer, 2001; Stamford et al., 2002; Cheng et al., 2017). Inoculation of SOB has been reported to enhance the growth and yield of different crop plants such as groundnut by 11% (Anandham et al., 2007), mustard by 6.6% (Chaudhary et al., 2017), onion by 45-50% (Awad et al., 2011), and mustard by 14.50–30.60% (Abhijit et al., 2014). We earlier reported the isolation of SOB from mud, coal, and drainage waters collected from open cost coal mines in India. Strains of *Stenotrophomonas maltophilia* and *S. pavanii* were identified to be most efficient for promotion of plant growth and sulfur nutrition in pigeonpea (Malviya et al., 2022). The detailed study was required to study the mechanisms by which S is transported from soil to roots and to shoots need to be deciphered.

Root colonization is an important attribute for any of the inoculant strains and provides clue for a commensal association between the two partners mediated through root exudates (Bais et al., 2006). *S. maltophilia* and *S. pavanii* were found to be good colonizers and formed biofilm on the root system. It has been reported that during the plant–microbe interaction, the expression of several genes of both plant and bacterial origin is modulated (Beauregard et al., 2013; de Souza et al., 2015). The bacterial genes associated with exo-polysaccharide production and biofilm formation are triggered by the root exudates during compatible interaction (Rudrappa et al., 2008; Meneses et al., 2011; Lopes et al., 2021). The formation of aggregates (micro-colonies) particularly by *S. maltophilia* on the roots indicates the copious production of EPS in the rhizosphere. It is worthwhile to mention that both *S. maltophilia* and *S. pavanii* form dry colonies on the growth medium. It has also been reported that the efficiency of bacteria in stimulating growth occurs in a density-dependent manner (Rudrappa et al., 2008). The stage at which the threshold level of microbial density is achieved, the biofilms work as a single unit to coordinate the release of molecules that helps in the promotion of plant growth through different mechanisms (McNear, 2013). A good colonization potential by both the SOB also gives an indication about rhizosphere competence as reported earlier (de Souza et al., 2015). This was further confirmed by the enhanced expression of *sox* genes in the treatments inoculated with SOB and S^6^ compound. Under sterile conditions, the enhanced expression of genes involved in S oxidation is directly related to population build-up of SOB. When *S. maltophilia* was inoculated along with S^6^ compound, the population build-up and root colonization were enhanced and the same was manifested in the higher expression of genes responsible for S-oxidation in the soil. Similar observations were made by Berben et al. (2019) and Zhang et al. (2019). The variations in the expression levels of *sox* genes in treatments where two different sources of S were amended (S^6^ or S^0^) irrespective of the inoculant strain indicate that the population build-up of SOB was higher in the presence of readily available source of sulfur (S^6^) as compared to elemental sulfur (S^0^).

The root system architecture (RSA) was also analyzed in both inoculated and uninoculated treatments. It is believed that the RSA is controlled by different biological and edaphic conditions (McNear, 2013). In this study, RSA was greatly influenced by inoculation of *S. maltophilia* and amendment of S^6^ compound. However, the same strain in presence of S^0^ could not influence the RSA to that extent. There are many reports regarding the modification of root architecture and anatomy in response to agriculturally important microorganisms so as to enhance the uptake of nutrients by the plants from the soil (Ortiz-Castro et al., 2008; Tian et al., 2014; Singh et al., 2017). It is well-known that larger root volume, root hair density, and increased number of lateral roots not only provide a better stand to the plant but also enhance the uptake and translocation of different nutrients from the soil to the plant (Singh et al., 2021).

Besides the root system architecture, the effect of inoculation of SOB and amendment of two different sources of sulfur (S^6^ and S^0^) was also studied at the enzymatic, non-enzymatic, and gene expression levels. The presence of unavailable form of sulfur (S^0^) is perceived by the plant as nutritional stress. In turn, the plant responds by regulating the antioxidative reaction and accumulation of polyphenolics in plant (Singh S. et al., 2020). Stress conditions, in general, accelerate the production of reactive oxygen species (ROS) in the plant system (Meng et al., 2016). To overcome the burst of ROS, the plants have developed both non-enzymatic (organic osmolyte like glycine betaine, proline, glutathione, etc.) and enzymatic (catalase, superoxide dismutase, ascorbate peroxidase, glutathione reductase, etc.) components (Nawaz and Wang, 2020; Singh D. P. et al., 2020). In this study, there was a significant increase in the accumulation of proline, flavonoids, total phenolics, and activities of antioxidant enzymes in the treatment amended with elemental sulfur and inoculated with SOB. The inoculation of SOB induced the synthesis of both enzymatic and non-enzymatic component which in turn provided protection to the plant from ROS. Similar results have been reported in different studies related to alleviation of abiotic stress by microbial inoculation (Singh et al., 2015, 2021). It is worthwhile to mention that microbial inoculants need to be developed that provide protection in the presence of elemental sulfur. Moreover, in different studies, use of S^0^ is recommended over that of sulfate, since it not only improves plant growth and nutrition but also increases systemic tolerance to different abiotic stresses (Degryse et al., 2016; Fuentes-Lara et al., 2019).

Sulfate transporters (*SULTRs*) are the key gene family responsible for the S-uptake and translocation in the higher plants. These are encoded by a large gene family, comprising of 12 genes in *Arabidopsis thaliana*, 10 in wheat (*Triticum* spp.), 12 in rice (*Oryza sativa*), 16 in Populus *(Populus stremula* × P. alba), and 28 in soybean (*Glycine max*). However, the literature is silent about the pigeonpea *SULTRs* and their role in S-nutrition. In-depth research is a pre-requisite to establish the relative contribution of the pigeonpea sulfate transporter genes to overall sulfate transport in plants. It is also necessary to explore whether all *SULTRs* are involved in sulfate acquisition, translocation, and remobilization of sulfur in the plant system. In this study, we performed a comprehensive investigation of the pigeonpea *SULTRs* gene family using comparative genomic and phylogenetic analyses. For this 10 *AtSULTRs*, 4 *GmSULTRs*, 9 *PsSULTRs*, 1 *OsSULTRs*, and 3 *TdSULTRs* were used as query sequences for BLASTn searches of the pigeonpea database (*Cajanus cajan*, taxid:3821) in NCBI with default parameters, and redundant sequences were discarded manually. Furthermore, qPCR analysis was done in the presence and absence of S-oxidizing bacteria in pigeonpea. This is the first report on the microbe-mediated induction of *PpSULTR* genes in pigeonpea and their role in S-uptake and translocation. A 7.56- to 27.33-fold changes in the expression of *PpSULTRs* were recorded at early crop growth stage (30 days after sowing), which is further confirmed by the enhanced sulfur content in the roots and shoots of pigeonpea. The expression of *SULTRs* in the plants supplemented with elemental S was significantly higher as compared to plants supplemented with S^6^ compounds at 30 days after sowing. Interestingly, the expression of *SULTRs* in the plants was significantly increased in the presence of potential SOB in the rhizosphere, suggesting their versatility in controlling *SULTRs* transcription.

**FIGURE 10:**
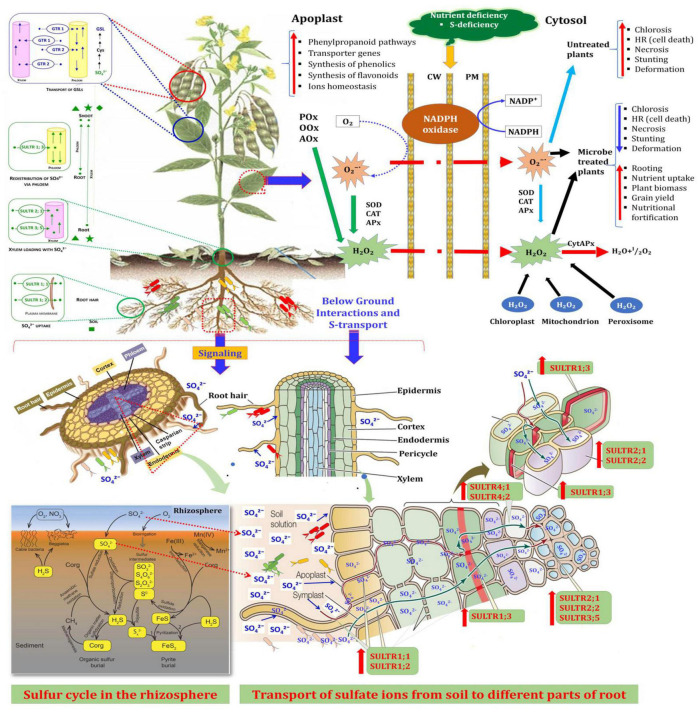
A comprehensive overview of plant-microbe interactions contributing to S-uptake in plants The oxidation of sulfur to sulfate by SOB in rhizosphere, its uptake by roots and its transport to shoots through involvement of S-transporter genes. The interaction results changes in the activity of radical scavenging enzymes and led to increase in growth and yield of pigeonpea.

This study provides the key evidence on molecular mechanism underlying microbial-induced expression of *SULTRs* in pigeonpea roots and shoots in the presence of the two possible enhancers, *S. maltophilia* DRC-18-7A and *S. pavanii* DRC-18-7B at an early stage of crop growth. Figure 10 depicts the possible interactions contributing to S-uptake in the plants. It is suggested that *S. maltophilia* DRC-18-7A and *S. pavanii* DRC-18-7B-dependent transcriptional induction and post-transcriptional regulation allow fine-tuning of the *SULTRs* transcript levels in roots and shoots of pigeonpea.

## Conclusion

The microorganisms and plant take up sulfur in the form of sulfate (S^6^). The elemental sulfur (S^0^) applied/present in the soil undergoes change in the oxidation state from S^0^ to S^6^ due to the action of specific group of bacteria collectively termed as sulfur-oxidizing bacteria. Inoculation of two potential SOB (*S. maltophilia* and *S. pavanii*) to pigeonpea led to the modifications in the root architecture that supports efficient uptake of nutrients. The enhanced activity of sulfur oxidation genes in inoculated treatments and *PpSULTR* genes in plants contributed to the enhanced uptake of sulfur in roots and shoots of pigeonpea. The increase in non-enzymatic and enzymatic components to counter ROS due to inoculation also contributed to the enhanced growth of pigeonpea. SOB with additional plant growth-promoting attributes could be recommended as potential inoculants for pigeonpea for commercial production after extensive field evaluation.

## Data availability statement

The datasets presented in this study can be found in online repositories. The names of the repository/repositories and accession number(s) can be found in the article/Supplementary material.

## Author contributions

DM, AS, US, and AV conceived and designed the experiments. DM, SS, and US performed the experiments. DM, AS, and US analyzed the data. DM and US wrote the manuscript. AS and AV have edited and given final touch to the manuscript. All authors have reviewed the manuscript and have given approval to the final version.

## Footnotes

1
http://au.expasy.org/tools/pi_tool.html

2
https://bioinfo.ut.ee/primer3-0.4.0/

3
https://www.ncbi.nlm.nih.gov/tools/primer-blast/index.cgi?LINK_LOC=BlastHome
