# Supplementary material for: Unraveling the mechanism of sulfur nutrition in pigeonpea inoculated with sulfur-oxidizing bacteria
Source: Front Microbiol. 2022 Sep 5;13:927702. doi: 10.3389/fmicb.2022.927702 (PMC9483215; doi:10.3389/fmicb.2022.927702)
Supplement: Supplementary file 1 [file Data_Sheet_1.docx]

**Supplementary Table 1.** Composition of nutrient solution used in this study

| **S. No.** | **Ingredient** | **Quantity used** |
| --- | --- | --- |
| **Nutrient solution with sulfate** | | |
|  | Calcium Phosphate (CaHPO_4_) | 1000 mg |
|  | Ammonium nitrate (NH_4_NO_3_) | 1650 mg |
|  | Potassium phosphate (KH_2_PO_4_) | 170 mg |
|  | Magnesium sulfate (MgSO_4_.7H_2_O) | 370 mg |
|  | Potassium nitrate (KNO_3_) | 1900 mg |
|  | Sodium chloride (NaCl) | 100 mg |
|  | Ferric chloride (FeCl_3_) | 100 mg |
|  | Boric acid (H_3_BO_3_) | 6.2 mg |
|  | Manganese sulfate (MnSO_4_.H_2_O) | 22.3 mg |
|  | Zinc sulfate (ZnSO_4_) | 8.6 mg |
|  | Sodium molybdate (Na_2_MoO_4_.2 H_2_O) | 0.25 mg |
|  | Potassium iodide (KI) | 0.83 mg |
|  | Copper (II) chloride | 0.025 mg |
| **Nutrient solution without sulfate ions** | | |
|  | Calcium Phosphate dibasic (CaHPO_4_) | 1000 mg |
|  | Ammonium nitrate (NH_4_NO_3_) | 1650 mg |
|  | Potassium phosphate (KH_2_PO_4_) | 170 mg |
|  | Magnesium chloride (MgCl_4_.7H_2_O) | 370 mg |
|  | Potassium nitrate (KNO_3_) | 1900 mg |
|  | Sodium chloride (NaCl) | 100 mg |
|  | Ferric chloride (FeCl_3_) | 100 mg |
|  | Boric acid (H_3_BO_3_) | 6.2 mg |
|  | Manganese chloride (MnCl_2_.H_2_O) | 22.3 mg |
|  | Zinc chloride (ZnCl_2_) | 8.6 mg |
|  | Sodium molybdate (Na_2_MoO_4_.2 H_2_O) | 0.25 mg |
|  | Potassium iodide (KI) | 0.83 mg |
|  | Copper (II) chloride (CuCl_2_) | 0.025 mg |
|  | Elemental S* | 1. mg |

* Elemental S was mixed in the Leonar jar containing sterile sand (500g) during experimentation.

**Supplementary Table 2.** Primers sequences of key gene involved in the sulfur oxidation in the *Stenotrophomonas* spp. used in semi-quantitative PCR analysis

| S.No. | Gene | Forward | Reverse |
| --- | --- | --- | --- |
|  | *Sulfide:quinine oxidoreductase (sqr)* | GCTGAGCAGCCTCAATCC | ACTCGGACCGCCGTTACTG |
|  |  | TCTCGGCAGCCAAAATGC | ACTCGGGAGACTCAATAG |
|  |  | CTGCTGCATCCTCAATAC | CCACGGCAGCGCCAATAC |
|  | *Sulfur oxygenas ereductase (sorAB)* | AAGCCCGTGCCTAAAGTG | CTGCCATGGATGATGTCGT |
|  |  | AAGCCCGTGCCTAAAGTG | ATGCCGGCGTTGGTGTTGG |
|  | *Tetrathionate hydrolase (tetH)* | AGCGCGACACGCTACCGG | GGCCGCTCAATGATAACC |
|  |  | AGGCCGACTCGCTACCCG | GGCCGCTCAATGATAACC |
|  | *Sulfur oxidizing enzyme (soxZ-I)* | ACCAGGGCAAGGTGATTC | CGTGGTGGCCTTCCAGGTC |
|  |  | AGGTGGACAAGTTGATTC | GTTGTTGCCGTTCCAGGTC |
|  | *Sulfur oxidizing enzyme (soxZ-II)* | CAGCTGGGAATGGCACTG | CAAAGATAAGGCTGGAAAA |
|  |  | TCATGGGCAAAGGCATTG | TGCAGAAAAGGCTGGAAAC |
|  |  | CTTCGGGCATTGGCAATG | ACAAGATAAGCCGGGAAAT |
|  | *sulfur oxidizing enzyme (soxA-II)* | ACGCTGATGCCGTTGGTG | TTACCATTTCCCCTCTTAT |
|  |  | CTGGCGATGCCGTGGCTG | CACCATTTCACGACTGAT |
|  | *Heterodisulfide Reductase (TQO)* | CCACCCAGGACTGCAGAA | CGGCCCAGGACGCCACCA |
|  | *Heterodisulfide Reductase A (hdrA)* | GCGAGGTGTAGGTGAAGC | CGGCGGCGACCATCTTTT |
|  | *Sulfur oxidizing enzyme (soxX-I)* | CAAGGGTTTTTGTTTGGC | GAACTTCAGCTGCGGGAA |
|  | *Sulfur oxidizing enzyme (soxY-II)* | CCATGCGTTTTTCGATGC | TGGCCACCATCGCTGAAAC |
|  | *Sulfur dioxygenase A (sdoA)* | TTTGTCCTGCTGCTGGGGGTGC | CCGTTGATGAGGATGTCT |
|  |  | TTGATCGTGCTGCTGGGCGTGC | CCGTTGATCAGGATGTCT |
|  | *Sulfur dioxygenase B (sdoB)* | CTCATGTTGCCCGAGGACAC | ACCGCT-GGTGGATGTGTTG |
|  |  | GTCACCTTGCCGGTGGAGCC | AGGGCGATGTAGATGTGTTT |
|  | *Thiosulfohydrolase (soxB)* | TGCGTCCGCCACGATGTAAT | ACTGCAGATCCATCTGTTTG |
|  | *Thiosulfate dihydrogenase (TsdA)* | CAGCACCAGCAGGAACGCCG | GCGCCCCGCGCTGGCCCCAA |
|  |  | GGGGACCACGATCAACGCCG | GGTCAGCGTGCCGGTGCCCA |
|  | *Sulfate adenylyltransferase (SAT)* | GTGAGGCCCTGGCCGAAGC | GCACGTCACCGAGCAGGAAGCG |
|  |  | GTGATGGCCTGGCCGATGC | GCAGGTCACCGGCCTGGACGCG |
|  | *rpoD* (Housekeeping gene) | ATGTACATGCGCGAAATGG | ATCGGTCAGATCGTTGAAGC |

**Supplementary Table 3.** Accession numbers of SULTR protein sequences used in this study

| Species | Protein name | Protein Accession (GenBank) |
| --- | --- | --- |
| *Glycine max* | *GmSULTR1;1a* | XP_003526594 |
| *Glycine max* | *GmSULTR1;1b* | XP_003526594 |
| *Glycine max* | *GmSULTR1;2a* | XP_003532966 |
| *Glycine max* | *GmSULTR1;2b* | XP_003547605 |
| *Glycine max* | *GmSULTR1;3a* | XP_003543770 |
| *Glycine max* | *GmSULTR1;3b* | XP_006596866 |
| *Glycine max* | *GmSULTR2;1a* | XP_003531364 |
| *Glycine max* | *GmSULTR2;1b* | XP_003538517 |
| *Glycine max* | *GmSULTR2;1c* | XP_003552820 |
| *Glycine max* | *GmSULTR2;2a* | XP_003526596 |
| *Glycine max* | *GmSULTR2;2b* | XP_003544185 |
| *Glycine max* | *GmSULTR2;2c* | XP_003543772 |
| *Glycine max* | *GmSULTR2;3* | XP_006601860 |
| *Glycine max* | *GmSULTR3;1a* | XP_003521258 |
| *Glycine max* | *GmSULTR3;1b* | XP_003554265 |
| *Glycine max* | *GmSULTR3;2a* | XP_003518908 |
| *Glycine max* | *GmSULTR3;2b* | XP_003536673 |
| *Glycine max* | *GmSULTR3;3a* | XP_003529415 |
| *Glycine max* | *GmSULTR3;3b* | XP_003556073 |
| *Glycine max* | *GmSULTR3;3c* | XP_003528541 |
| *Glycine max* | *GmSULTR3;4a* | XP_003529722 |
| *Glycine max* | *GmSULTR3;4b* | XP_003531685 |
| *Glycine max* | *GmSULTR3;4c* | XP_003543650 |
| *Glycine max* | *GmSULTR3;4d* | XP_003546346 |
| *Glycine max* | *GmSULTR3;5a* | XR_136691 |
| *Glycine max* | *GmSULTR3;5b* | XR_416059 |
| *Glycine max* | *GmSULTR4;1* | XP_003520027 |
| *Glycine max* | *GmSULTR4;2* | XP_003552670 |
| *Arabidopsis* *thaliana* | *AtSULTR1;1* | AB018695 |
| *Arabidopsis* *thaliana* | *AtSULTR1;2* | AB042322 |
| *Arabidopsis* *thaliana* | *AtSULTR1;3* | AB049624 |
| *Arabidopsis* *thaliana* | *AtSULTR2;1* | AB003591 |
| *Arabidopsis* *thaliana* | *AtSULTR2;2* | D85416 |
| *Arabidopsis* *thaliana* | *AtSULTR3;1* | D89631 |
| *Arabidopsis* *thaliana* | *AtSULTR3;2* | AB004060 |
| *Arabidopsis* *thaliana* | *AtSULTR3;3* | AB023423 |
| *Arabidopsis* *thaliana* | *AtSULTR3;4* | AB054645 |
| *Arabidopsis* *thaliana* | *AtSULTR3;5* | AB061739 |
| *Arabidopsis* *thaliana* | *AtSULTR4;1* | AB008782 |
| *Arabidopsis* *thaliana* | *AtSULTR4;2* | AB052775 |
| *Oryza sativa* | *OsSULTR1;1* | AF493790 |
| *Oryza sativa* | *OsSULTR1;2* | XP_470587 |
| *Oryza sativa* | *OsSULTR1;3* | AF493790 |
| *Oryza sativa* | *OsSULTR2;1* | AAN59769 |
| *Oryza sativa* | *OsSULTR2;2* | AAN59770 |
| *Oryza sativa* | *OsSULTR3;1* | NP_921514 |
| *Oryza sativa* | *OsSULTR3;2* | AAN06871 |
| *Oryza sativa* | *OsSULTR3;3* | AK104831 |
| *Oryza sativa* | *OsSULTR3;4* | BAD68396 |
| *Oryza sativa* | *OsSULTR3;5* | NM_192602 |
| *Oryza sativa* | *OsSULTR3;6* | NM_191791 |
| *Oryza sativa* | *OsSULTR4;1* | AF493793 |
| *Cajanus cajan* | *CcSULTR2.0* | XM_029272652 |
| *Cajanus cajan* | *CcSULTR2.0* | XM_020367167 |
| *Cajanus cajan* | *CcSULTR3.0* | XM_020380400 |
| *Cajanus cajan* | *CcSULTR3-like* | XM_029272617 |
| *Cajanus cajan* | *CcSULTR3-like* | XM_029269635 |
| *Cajanus cajan* | *CcSULTR3-like* | XM_029269241 |
| *Cajanus cajan* | *CcSULTR3.0* | XM_020374332 |
| *Cajanus cajan* | *CcSULTR3.1* | XM_020353231 |
| *Cajanus cajan* | *CcSULTR3.4* | XM_029273486 |
| *Cajanus cajan* | *CcSULTR1.3* | XM_020349173 |
| *Cajanus cajan* | *CcSULTR1.3* | XM_020349181 |
| *Cajanus cajan* | *CcSULTR1.3* | XM_020349166 |
| *Cajanus cajan* | *CcSULTR1.3* | XM_020349158 |
| *Cajanus cajan* | *CcSULTR2.1* | XM_020359338 |
| *Cajanus cajan* | *CcSULTR2.1* | XM_029270232 |
| *Cajanus cajan* | *CcSULTR3.1* | XM_020353230 |
| *Cajanus cajan* | *CcSULTR3.3* | XM_029269081 |
| *Cajanus cajan* | *CcSULTR3.3* | XM_020346255 |
| *Cajanus cajan* | *CcSULTR2.1* | XM_020381351 |
| *Cajanus cajan* | *CcSULTR3.1* | XM_020380658 |
| *Cajanus cajan* | *CcSULTR3.4* | XM_020379899 |
| *Cajanus cajan* | *CcSULTR3.5* | XM_020377651 |
| *Cajanus cajan* | *CcSULTR4.2* | XM_020376339 |
| *Cajanus cajan* | *CcSULTR1.3* | XM_029273853 |
| *Cajanus cajan* | *CcSULTR1.3* | XM_029273852 |
| *Cajanus cajan* | *CcSULTR3.4* | XM_020372579 |
| *Pisum sativum* | *PsSULTR1.1-like* | KP759563 |
| *Pisum sativum* | *PsSULTR1.2-like* | KP759564 |
| *Pisum sativum* | *PsSULTR1.3-like* | KP759565 |
| *Pisum sativum* | *PsSULTR2.2-like* | KP759566 |
| *Pisum sativum* | *PsSULTR3.1-like* | KP759567 |
| *Pisum sativum* | *PsSULTR3.1-like* | KP759568 |
| *Pisum sativum* | *PsSULTR3.3-like* | KP759570 |
| *Pisum sativum* | *PsSULTR3.4-like* | KP759571 |
| *Pisum sativum* | *PsSULTR3.5-like* | KP759572 |
| *Pisum sativum* | *PsSULTR4.1* | KP759573 |
| *Triticum durum* | *TdSULTR1.1* | JX896648 |
| *Triticum durum* | *TdSULTR1.3* | JX896649 |

**Supplementary Table 4.** Oligonucleotide primer sequences of *PpSULTR* genes used in qPCR expression analysis

| **S.N.** | **Gene** | | **Forward primer** | | **Reverse primer** | |  |
| --- | --- | --- | --- | --- | --- | --- | --- |
|  | | *PREDICTED: Cajanus cajan sulfate transporter 1.1* | | CTTGGCATTGAAGCCATTTT | | CCACTGCCACTGGTCCTATT | |
|  |  |  |  | GTTGCAGAGGCTGTTGCATA | | TAGCACCAACCAGCACAAAG | |
|  | | *PREDICTED: Cajanus cajan sulfate transporter 1.2* | | GCATCCAAGCCTTTGAAGAG | | CCTCAACCACCTTTGGAGAA | |
|  | | *PREDICTED: Cajanus cajan sulfate transporter 1.3* | | GGTCCTCCTGCTGATGAAAA | | CCTCAATACCGAGGATGAGC | |
|  | | *PREDICTED: Cajanus cajan high affinity sulfate transporter 2.2* | | GCTTTCATGGGAAGCTCAAG | | TGGCAGCATGAGACAAAAAG | |
|  |  |  |  | CCTTCTAATTGCGGTGGGTA | | GGGCTTCTTCATCTGTCAGC | |
|  |  |  |  | ATGCGATCGGTTTTCAATTC | | TGACACGCCTTTCTTGTCTG | |
|  |  |  |  | TGTCCCTCTTGCTTGGAACT | | CCAAGCAGACCCTTAAGCTG | |
|  |  |  |  | CCTTCTAATTGCGGTGGGTA | | CCTTTGGGCTTCTTCATCTG | |
|  | | *PREDICTED: Cajanus cajan high affinity sulfate transporter 2.1* | | GGGAAATTCAAGAAGCACCA | | CCGATTTTGCCAACAAAGTT | |
|  |  |  |  | ATCCTTGGAACCCTCGAAAT | | CTGATGGATGGAGCTTGGAT | |
|  |  |  |  | TTGGGGTTCATTGTGGATTT | | CAGGACAAAATTGCGAGGAT | |
|  |  |  |  | GACGTGCCCTTCAAAACATT | | ATTGAGCACCCACTGAGACC | |
|  | | *PREDICTED: Cajanus cajan low affinity sulfate transporter 3* | | CTGGTCCCAAAAGTGGAAGA | | CCTTTGAGCTGCTGAAGACC | |
|  |  |  |  | CTGGTCCCAAAAGTGGAAGA | | CCCTTTGAGCTGCTGAAGAC | |
|  |  |  |  | TTTCAGGTTGGGGTTTCTTG | | AGGAATGAACAGCCGATGAC | |
|  |  |  |  | CAGAAGCTCAAAGTGGCACA | | AGTGGGCACAATCACAAACA | |
|  | | *PREDICTED: Cajanus cajan low affinity sulfate transporter 3-like* | | ACTGGGTTGAATGCTTTTCG | | AGGACCGCTGTGACAGAGAC | |
|  |  |  |  | TTGCCATGTTGAAGGAGAAA | | ACCACGTGAAAGCAACCTCT | |
|  |  |  |  | GCCAGACTTTGCAGTGTGAG | | CGCCTTAGAGCTTCGGTATG | |
|  |  |  |  | CCACACCCCTTCTCTCTTCA | | TCAGTGAGCAGCTTGAGGAA | |
|  |  |  |  | TGCCAATGGTGGATTACAGA | | ACCACGTGAAAGCAACCTCT | |
|  |  |  |  | TGCCAATGGTGGATTACAGA | | TACACCACGTGAAAGCAACC | |
|  | | *Cajanus cajan sulfate transporter 3.1* | | TGACCGAGTCATATGCCAAA | | GACCCGTGCATCTTTCATTT | |
|  |  |  |  | CCTCTGGTGGTTCTTTCAGC | | AACAAATGTCCTTGGCCTTG | |
|  |  |  |  | CCATTGCTAGTTTGGCCATT | | AGTGAAAGCGAGGTGGAGAA | |
|  |  |  |  | AAGCTACAGCGCAAACCAGT | | GCTCCAACAGCCTCTTCAAC | |
|  | | *PREDICTED: Cajanus cajan probable sulfate transporter 3.3* | | TTTCTCATGCCATTGTTCCA | | CCCAAGAAAGCAACCAACAT | |
|  |  |  |  | AGCAATGATGCCACACGTAA | | GGAAAATTAGGCCCCACAAT | |
|  |  |  |  | TTGCTGGGATCTTTCAATCC | | TTTGCCATGACCACTCGTTA | |
|  |  |  |  | TGAAGGGTGTTGAGCTTGTG | | TTGGTGATTGACCCTCCTTC | |
|  | | *PREDICTED: Cajanus cajan probable sulfate transporter 3.4* | | TGGAAAGCTCAGCCTTGATT | | TCCCTCTAGCAAAAGCCAAA | |
|  |  |  |  | AATCCAAACCATGCCATCAT | | TGAAGTGCGAGGAGGAACTT | |
|  |  |  |  | AACGGGATCCTTTTCTCGAT | | GATGATAATGGCCGCTAGGA | |
|  | | *PREDICTED: Cajanus cajan probable sulfate transporter 3.5* | | GTAACGCCGAGGTTCACAAT | | CATCACCCTCCTCGATGTCT | |
|  |  |  |  | ATTTTTGGCGCCACTATTTG | | GAACCCCAAGTCCAACAGAA | |
|  | | *PREDICTED: Cajanus cajan sulfate transporter 4.1* | | TTGGCTTCCCACCATTTTCT | | CAGCCAACACCAAGACCAAC | |
|  | | *PREDICTED: Cajanus cajan probable sulfate transporter 4.2* | | GCTGCTTCGTTTCATTAGCC | | TCGATTTTCCCAGGTGTTTC | |

**Supplementary Table 5.** Primers sequences of key gene involved in the phenylpropanoid pathways in pigeon pea and used for SYBR Green real time qPCR expression analysis

| **S.N.** | **Gene** | **Forward primer** | **Reverse primer** |
| --- | --- | --- | --- |
|  | *Phenylalanine ammonia-lyase* [EC:4.3.1.24] | TGGCTTTGAGAGACGGAGTT | TCCCAATCTTTGAGGCATTC |
|  | *4-coumarate-CoA ligase* [EC:6.2.1.12] | GACCCAGAAGCAACTTCAGC | GGAATAACTGCGGCATCAAT |
|  | *Cinnamoyl-CoA reductase* [EC:1.2.1.44] | TTATCGCCTCTTGGATGGTC | GGGTTATCGGTGACAGGAGA |
|  | *Cinnamyl-alcohol dehydrogenase* [EC:1.1.1.195] | GGAAATGAAGGCATTGGCTA | CCTTTGCAGCACAAAAGTCA |
|  | *5-O-(4-coumaroyl)-D-quinate 3'-monooxygenase* [EC:1.14.14.96] | CCGGTTGTCGGTAACCTCTA | TTCCCGTCTCTGCTGAACTT |
|  | *Caffeoylshikimate esterase* [EC:3.1.1.-] | GCCTCCCTCTCATTCTTCCT | CCAAACAAGAGGCCGTACAT |
|  | *Ferulate-5-hydroxylase* [EC:1.14.-.-] | AGGGAGCAACTTCGAGTTCA | TGAGTCCGAACACGTCACTC |
|  | *Caffeoyl-CoA O-methyltransferase* [EC:2.1.1.104] | TCTGATCGGGTACGACAACA | ACCTACAGGAAGCTGGCAGA |
|  | *Caffeic acid 3-O-methyltransferase* [EC:2.1.1.68 2.1.1.4] | TGCTGGCTCACTAGGTCCTT | AATTAAAGTCGCACGGTTGG |
|  | *Peroxidase* [EC:1.11.1.7] | CCCCACTTTATCCGTCTTCA | CTGTCTGCACACCCAAGAGA |
